# Impact of Coronary Artery Disease Severity Assessed With the SYNTAX Score on Outcomes Following Transcatheter Aortic Valve Replacement

**DOI:** 10.1161/JAHA.116.005070

**Published:** 2017-02-20

**Authors:** Jean‐Michel Paradis, Jonathon M. White, Philippe Généreux, Marina Urena, Darshan Doshi, Tamim Nazif, Rebecca Hahn, Isaac George, Omar Khalique, Kishore Harjai, Laura Lasalle, Benoit M. Labbé, Robert DeLarochellière, Daniel Doyle, Éric Dumont, Siamak Mohammadi, Martin B. Leon, Josep Rodés‐Cabau, Susheel Kodali

**Affiliations:** ^1^ Quebec Heart and Lung Institute Quebec Canada; ^2^ Cardiovascular Research Foundation New‐York NY; ^3^ Hopital du Sacre-Coeur de Montreal Montreal Quebec Canada; ^4^ Gagnon Cardiovascular Institute Morristown Medical Center Morristown New Jersey; ^5^ Columbia University Medical Center New‐York NY

**Keywords:** aortic stenosis, coronary artery disease, SYNTAX score, transcatheter aortic valve replacement, Aortic Valve Replacement/Transcather Aortic Valve Implantation, Catheter-Based Coronary and Valvular Interventions, Revascularization, Valvular Heart Disease

## Abstract

**Background:**

The influence of coronary artery disease (CAD) on clinical and echocardiographic outcomes after transcatheter aortic valve replacement (TAVR) is still controversial. We sought to evaluate the impact of CAD severity as measured by the SYNTAX score (SS) on patients undergoing TAVR.

**Methods and Results:**

A total of 377 patients who underwent TAVR in 2 high‐volume centers in North America were included in our retrospective analysis. A blinded angiographic core laboratory calculated the SS on all available coronary angiograms with the use of quantitative coronary analysis. Patients were stratified into 4 groups: (1) no CAD (SS=0); (2) low SS (SS between 1 and 22); (3) intermediate SS (SS between 23 and 32); and (4) high SS (SS ≥33). Patients who had undergone percutaneous coronary intervention within 6 months prior to TAVR were separated into 2 categories based on their residual SS (<8 and ≥8). Patients with previous coronary artery bypass grafting (CABG) were divided into 2 groups: (1) low CABG SS and (2) high CABG SS. The primary end point was a composite of all‐cause mortality, myocardial infarction, and stroke. At 30 days and 1 year, both the presence and the severity of CAD had no impact on the rate of the combined primary end point and on all‐cause mortality, cardiovascular mortality, and myocardial infarction. Patients with less complete revascularization (residual SS ≥8 versus residual SS <8 and low CABG SS versus high CABG SS, had similar rates of the combined primary end point, all‐cause mortality, cardiovascular mortality, MI, and stroke, at both 30 days and 1 year.

**Conclusions:**

In our core laboratory–validated study, neither the severity of CAD nor completeness of revascularization after percutaneous coronary intervention or CABG were associated with clinical outcomes after TAVR, at both 30 days and 1 year.

## Introduction

Aortic valve stenosis is the most frequent valvular heart disease in the elderly population.^1^, ^2^, ^3^ Atherosclerosis and aortic stenosis (AS) share common risk factors.^4^ Indeed, histopathologic data show that degenerative calcific AS is a complex disease process similar to atherosclerosis and elastocalcinosis.^5^ Therefore, patients with severe AS often have concomitant significant coronary artery disease (CAD).^6^ The risk of surgical aortic valve replacement is increased in the presence of CAD, and coronary revascularization with coronary artery bypass grafting (CABG) is generally recommended at the time of surgery.^3^, ^7^, ^8^ In the era of transcatheter aortic valve replacement (TAVR), treatment paradigms have shifted. Moreover, among patients with severe AS, the severity and complexity of CAD is heterogeneous. Consequently, a lot of questions remain unanswered regarding the impact of coronary disease and its optimal management in the setting of TAVR. The SYNTAX score (SS) has been shown to be useful not only to evaluate the extent of CAD, and therefore risk‐stratify patients with CAD, but also to predict clinical outcomes in various subsets of patients undergoing percutaneous coronary intervention (PCI).^9^ This study seeks to evaluate the influence of CAD severity as measured by the SS on patients with severe symptomatic AS undergoing TAVR.

## Methods

### Patient Population

All consecutive patients who underwent TAVR between May 2007 and December 2012 in 2 high‐volume centers in North America (Columbia University Medical Center, New York, and Quebec Heart and Lung Institute, Quebec, Canada) with the Edwards SAPIEN, SAPIEN XT, or SAPIEN 3 transcatheter heart valves (Edwards Lifesciences, Irvine, CA) were included in this study. A multidisciplinary team composed of interventional cardiologists and cardiac surgeons established the eligibility of patients for TAVR based on recognized risk scores (Society of Thoracic Surgeons, Logistic EuroSCORE), anatomical considerations, and clinical judgment. Individuals deemed eligible for TAVR underwent a systematic workup that included echocardiography, coronary angiography, aorto‐iliofemoral angiography, and multidetector computed tomography. Patients were then selected for a transfemoral or transapical approach depending on the size, tortuosity, and degree of calcification of the iliofemoral arteries.

With regard to coexisting CAD, the treatment strategy and completeness of revascularization was decided by heart team consensus, taking into account coronary artery size, technical complexity of PCI, the amount of myocardium at risk, and the mode of presentation (eg, acute coronary syndrome). Chronic total occlusions and lesions in small vessels were not considered for revascularization. When PCI was elected, it was performed through transradial or transfemoral access and the use of drug‐eluting stents or bare metal stents was left to the operator's judgment. Dual antiplatelet therapy with aspirin and clopidogrel was strongly recommended for at least 1 month after bare metal stent implantation and at least 1 year following drug‐eluting stent placement. The study was approved by local ethics committees. All patients provided written informed consent for the procedures.

### Angiographic Evaluation

All available coronary angiograms were analyzed at the Angiographic Core Laboratory (core lab) at the Cardiovascular Research Foundation (New York, USA). Since one limitation of the SS is its dependence on angiographic interpretation, which can result in overestimation or underestimation of lesion severity,^10^ all angiograms were analyzed with the use of quantitative coronary analysis (QCA), which has been shown to be superior to visual estimates of vessel size.^11^ Through digitalization, image calibration, and arterial contour detection, QCA allowed the calculation of a precise QCA‐derived SS. CAD was defined as a ≥50% diameter stenosis by QCA estimation in vessels ≥1.5 mm in diameter.^9^ Angiographic core lab staff were blinded to all clinical outcomes. Interobserver reproducibility for the SS was 0.84 (kappa; 95% CI, 0.76–1.00).^12^ For patients who had undergone PCI within 6 months prior to TAVR, the residual SS (rSS), an objective measure of the degree and complexity of residual stenosis after PCI, was computed to assess the extent of CAD at the time of TAVR.^13^ Patients with an rSS of 0 were defined as having undergone complete revascularization. For patients with prior coronary artery bypass surgery (CABG), the CABG SS was calculated and reviewed by 2 interventional cardiologists (J.M.P. and D.D.) who were also blinded to clinical outcomes.

### Study End Points

The primary end point was a composite of all‐cause mortality, myocardial infarction (MI), and stroke. Secondary end points included death from cardiovascular cause, MI, repeat hospitalization, New York Heart Association functional class, vascular complications, major bleeding, 6‐minute walk distance, left ventricular ejection fraction, and valve performance (as assessed by echocardiography). All end points were adjudicated according to the following Valve Academic Research Consortium I definitions^14^ or according to the definitions established in the PARTNER 1 (Placement of Aortic Transcatheter Valves) trial protocol.^15^ Cardiovascular death was defined as any death caused by proximate cardiac cause (eg, MI, cardiac tamponade, worsening heart failure), unwitnessed death and death of unknown cause, all procedure‐related deaths including those related to a complication of the procedure or treatment for a complication of the procedure, death caused by noncoronary vascular conditions such as cerebrovascular disease, pulmonary embolism, ruptured aortic aneurysm, dissecting aneurysm, or other vascular disease. Periprocedural MI was defined as new ischemic signs or symptoms or new ischemic signs and elevated cardiac biomarkers (preferably creatine kinase‐MB) within 72 hours after the index procedure, consisting of two or more postprocedure samples 6 to 8 hours apart with a 20% increase in the second sample and a peak value exceeding 10 times the 99th percentile upper reference limit, or a peak value exceeding 5 times the 99th percentile upper reference limit with new pathological Q waves in at least 2 contiguous leads. Spontaneous MI was diagnosed in case of detection of rise and/or fall of cardiac biomarkers with at least one value above the 99th percentile upper reference limit, together with evidence of myocardial ischemia with at least one of the following: ECG changes indicative of new ischemia, new pathological Q waves in at least two contiguous leads, imaging evidence of new loss of viable myocardium, or new wall motion abnormality. Major stroke was defined as rapid onset of a focal or global neurological deficit persisting for more than 24 hours or mandating a therapeutic intervention, without another readily identifiable nonstroke etiology, and with confirmation of the diagnosis by a neurologist or by a neuroimaging modality (magnetic resonance imaging or computed tomography scan or cerebral angiography). Major vascular complication was determined in case of thoracic aortic dissection; access site or access‐related vascular injury leading to either death, need for significant blood transfusion (≥4 U), unplanned percutaneous or surgical intervention, or irreversible end organ damage; or distal embolization (noncerebral) from a vascular source requiring surgery or resulting in amputation or irreversible end organ damage. Stage 3 acute kidney injury was defined as an increase in serum creatinine to ≥300% of baseline (>3× increase from baseline) or serum creatinine of ≥4.0 mg/dL (≥354 μmol/L) with an acute increase of at least 0.5 mg/dL (44 μmol/L). In both centers, patients were followed during the index hospitalization and at 30 days, 6 months, 1 year, and yearly thereafter.

In both centers, adverse events were evaluated in the hospital, and by means of follow‐up visits at 1, 6, and 12 months.

### Statistical Analysis

Patients were stratified into 4 groups based on the presence and severity of CAD: (1) no CAD (SS=0); (2) low SS (SS between 1 and 22); (3) intermediate SS (SS between 23 and 32); and (4) high SS (SS ≥33). Based on previous studies showing a worst long‐term clinical prognosis in individuals with rSS >8,^13^, ^16^ 1‐year outcomes were compared between patients with complete revascularization or low rSS (rSS <8) and patients with significant residual coronary disease (rSS ≥8). Patients with previous CABG were divided into 2 groups: (1) patients with a CABG SS lower than the median CABG SS value for our study (C1); and (2) patients with a CABG SS greater than or equal to the median value (C2).

Continuous variables were reported as mean (±SD) or median (interquartile range), where appropriate, and were compared using Wilcoxon rank sum test for medians. Categorical variables were presented as frequencies (percentages). Comparisons between groups were performed using the Pearson chi‐squared test for categorical variables except when minimum expected values in any of the cells of a contingency table was below 5. In such cases, Fisher exact test was performed. The Cochran‐Armitage test was used to test for trends. Survival curves for time‐to‐event variables were performed with the use of Kaplan–Meier estimates and were compared between groups with the use of the log‐rank test.

All statistical analyses were performed using SAS version 9.4 (SAS Institute, Cary, NC). A *P*<0.05 was considered statistically significant.

## Results

A total of 377 patients who underwent TAVR were included in our analysis. Of those, 82 had no CAD, 129 were in the low SS group (1–22), 48 were in the intermediate SS group (23–32), and 118 were in the high SS group (≥33). Baseline clinical, echocardiographic, and procedural characteristics are presented in Table 1. Higher SS was associated with male sex, history of smoking, hyperlipidemia, and previous MI. Furthermore, a trend was observed between higher SS and history of smoking (*P*_trend_=0.043), hyperlipidemia (*P*_trend_=0.046), and previous MI (*P*_trend_=0.004). Moreover, greater CAD severity at baseline was linked to higher logistic EuroSCORE, lower left ventricular ejection fraction (LVEF), and less complete revascularization prior to TAVR, as illustrated by higher rSS and superior CABG SS.

**Table 1:** Baseline Clinical, Echocardiographic, and Procedural Characteristics

	No CAD n=82	Low SS n=129	Intermediate SS n=48	High SS n=118	Total N=377	*P* Value
Age, y	82.9±6.4	83.0±8.8	81.5±9.2	82.1±7.1	82.5±7.9	0.59
Male, %	40.7	41.1	54.2	70.3	51.9	<0.0001
Hypertension, %	84.1	87.6	89.6	89.0	87.5	0.74
History of smoking, %	16.7	35.7	36.4	38.6	32.4	0.002
Hyperlipidemia, %	63.4	80.6	91.7	90.6	81.4	<0.0001
Diabetes mellitus, %	32.9	30.2	31.3	32.2	31.6	0.98
History of CHF, %	65.9	75.2	60.4	83.8	73.9	0.02
Atrial fibrillation, %	40.2	22.4	25.0	22.9	26.8	0.02
Permanent pacemaker, %	14.6	20.2	22.9	28.8	22.0	0.11
Previous MI, %	2.4	33.3	41.7	49.2	32.6	<0.0001
Previous PCI, %	2.4	61.2	62.5	40.7	42.2	<0.0001
Previous CABG, %	0	17.1	52.1	93.2	42.7	<0.0001
Cerebrovascular disease, %	14.3	18.0	17.0	20.6	17.8	0.75
Peripheral vascular disease, %	25.6	31.5	23.4	39.0	31.6	0.12
COPD, %	31.7	29.5	18.8	27.1	27.9	0.43
NYHA, %						
Class II	17.1	7.9	31.3	10.3	13.7	0.0004
Class III	73.2	75.4	58.3	70.9	71.3	0.16
Class IV	9.8	16.7	10.4	18.8	15.0	0.24
6MWT distance, m	169.5	151.0	195.5	173.7	167.9	0.18
Baseline creatinine, mg/dL	1.23	1.37	1.37	1.38	1.34	0.31
Hemodialysis at baseline, %	1.2	0.8	2.1	0.8	1.1	0.89
Mean systolic BP, mm Hg	126.9	126.3	119.6	123.1	124.6	0.11
Mean diastolic BP, mm Hg	67.2	62.6	59.9	61.2	62.8	0.008
Logistic EuroSCORE	19.8	22.3	23.3	33.4	25.4	<0.0001
STS risk score	7.3	8.6	7.1	9.6	8.5	0.005
SS, mean	···	11.5	27.7	45.9	24.1	<0.0001
Residual SS, mean	···	6.4	16.4	33.8	19.2	<0.0001
CABG SS, mean	···	6.2	12.2	32.5	15.5	<0.001
Porcelain aorta, %	15.9	18.5	25.5	7.9	15.5	0.02
Frailty, %	32.1	40.9	31.9	34.5	35.7	0.54
Mean LVEF, %	53.7	53.9	50.6	48.2	51.6	0.007
Peak gradient, mm Hg	68.7	70.5	66.3	63.9	67.5	0.21
Mean gradient, mm Hg	42.3	42.6	40.6	38.3	40.9	0.15
Aortic valve area, cm^2^	0.62	0.66	0.64	0.65	0.64	0.7
Approach, %						
Transapical	45.1	54.3	43.8	56.8	51.7	0.24
Transfemoral	54.9	45.7	56.2	43.2	48.3	0.24
Valve size, %						
23 mm	57.3	41.1	47.9	33.1	43.0	0.006
26 mm	39.0	55.0	50.0	59.3	52.3	0.02
29 mm	3.7	3.9	2.1	7.6	4.7	0.24
Procedural success, %	88.9	89.9	97.9	92.3	91.5	0.29

6MWT indicates 6‐minute walk test; BP, blood pressure; CABG, coronary artery bypass graft surgery; CAD, coronary artery disease; CHF, congestive heart failure; COPD, chronic obstructive pulmonary disease; LVEF, left ventricular ejection fraction; MI, myocardial infarction; NYHA, New York Heart Association; PCI, percutaneous coronary intervention; SS, SYNTAX score; STS, Society of Thoracic Surgeons.

**Figure 1:**
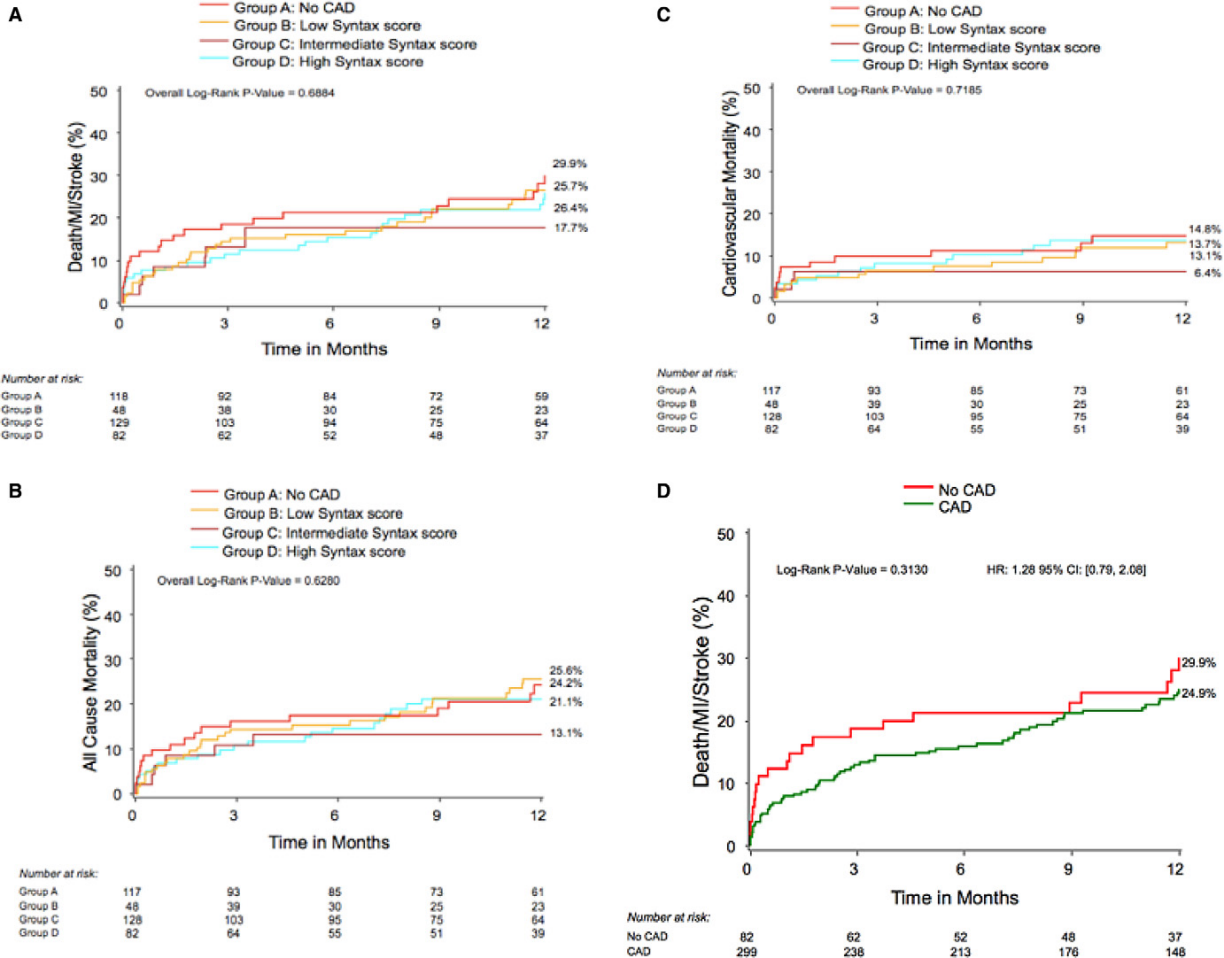
Kaplan–Meier curves at 1 year according to the SYNTAX score for (A) the combined end point (all cause death, myocardial infarction [MI], stroke); (B) all‐cause mortality; (C) cardiovascular mortality; and (D) Kaplan–Meier curves for the combined end point when comparing only 2 groups: no coronary artery disease (CAD) or CAD (regardless of the SYNTAX score). HR indicates hazard ratio.

At 30 days and 1 year, both the presence and the severity of CAD had no impact on the rate of the combined primary end point or on all‐cause mortality, cardiovascular mortality, and MI (Table 2 and Figure 1). On the contrary, the rate of stroke at 1 year was significantly different between the 4 subgroups of patients assessed in our study (no CAD [A]: 7.3%; low SS [B]: 0%; intermediate SS [C]: 2.1%; high SS [D]: 3.4% [*P*=0.02]). Indeed, the rate of stroke was significantly higher in patients without CAD than in patients in the low SS group (A versus B, *P*=0.0002). The presence and extent of CAD had no association with the 1‐year incidence of major vascular complication, major bleeding, repeat hospitalization, New York Heart Association class, or 6‐minute walk test distance.

**Table 2:** Outcomes According to Baseline SS (Chi‐Square) at 30 Days and 1 Year

	No CAD	Low SS	Intermediate SS	High SS	Total	*P* Value
30‐d outcomes						
Combined primary end point (death, MI, stroke)	13.4	7.0	10.4	9.3	9.5	0.48
All‐cause mortality	9.8	7.0	8.3	6.8	7.7	0.87
Cardiovascular mortality	7.3	4.7	6.3	4.3	5.3	0.78
MI	4.2	0	0	1.6	1.2	0.42
Stroke	4.9	0	2.1	2.6	2.1	0.12
1‐year outcomes						
Combined primary end point (death, MI, stroke)	26.8	23.3	16.7	22.0	22.8	0.61
All‐cause mortality	22.0	22.7	12.5	18.8	20.0	0.47
Cardiovascular mortality	13.4	10.9	6.3	12.0	11.2	0.64
MI	12.5	1.7	5.3	3.3	4.3	0.16
Stroke	7.3	0	2.1	3.4	2.9	0.02
Major vascular complications	13.4	4.7	12.5	3.4	7.2	0.01
Major bleeding	8.6	7.8	14.6	5.2	8.0	0.25
Repeat hospitalization	36.6	32.8	43.8	29.9	34.1	0.36
NYHA, %						
Class I	47.6	42.9	42.1	55.0	47.4	0.39
Class II	38.1	36.3	44.7	35.0	37.5	0.77
Class III	11.1	18.7	13.2	8.8	13.2	0.26
Class IV	3.2	2.2	0	1.3	1.8	0.67
6MWT distance, m	197.5	222.7	276.3	230.0	228.3	0.11

6MWT indicates 6‐minute walk test; CAD, coronary artery disease; MI, myocardial infarction; NYHA, New York Heart Association heart failure; SS, SYNTAX score.

Analysis of echocardiographic outcomes at 1 year showed that there was a significant trend toward lower LVEF with progression of CAD severity (A: 60.5%; B: 59.5%; C: 47.4%; D: 51.0% [*P*<0.0001]) (Table 3). However, when LVEF at 1 year was compared with LVEF at baseline (ΔLVEF), there was no statistically significant LVEF improvement in each of the 4 groups (Table 3). When patients were stratified by SS, there were no statistically significant differences in peak or mean aortic gradient, the presence of moderate or severe aortic regurgitation, or moderate or severe mitral regurgitation.

**Table 3:** Echocardiographic Outcomes at 1 Year

	No CAD	Low SS	Intermediate SS	High SS	Total	*P* Value
LVEF	60.5	59.5	47.4	51.0	55.6	<0.0001
Mean delta LVEF (1 y compared with baseline)	4.2	3.0	−2.8	0.3	1.7	0.1
Peak aortic gradient, mm Hg	22.5	20.3	21.5	18.5	20.3	0.05
Mean aortic gradient, mm Hg	12.1	10.7	11.7	10.0	10.9	0.08
AVA, cm^2^	1.43	1.51	1.52	1.60	1.52	0.08
Total moderate or severe AR, %	5.2	3.2	2.2	2.7	3.4	0.95
Moderate or severe MR, %	13.3	10.1	20.9	15.1	13.7	0.32

AVA indicates aortic valve area; AR, aortic regurgitation; CAD, coronary artery disease; LVEF, left ventricular ejection fraction; MR, mitral regurgitation; SS, SYNTAX score.

### Residual SS

**Figure 2:**
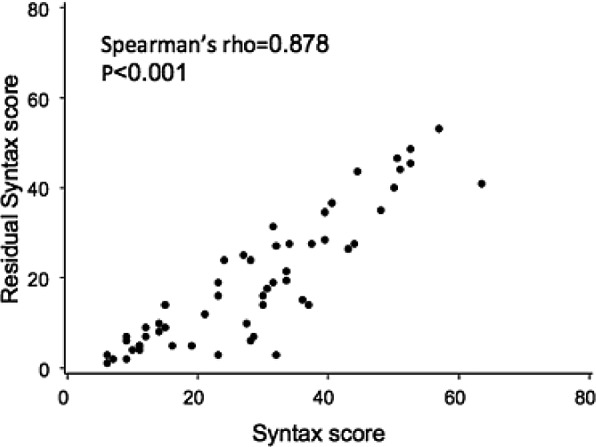
Correlation between the SYNTAX score at baseline and the residual SYNTAX score.

**Figure 3:**
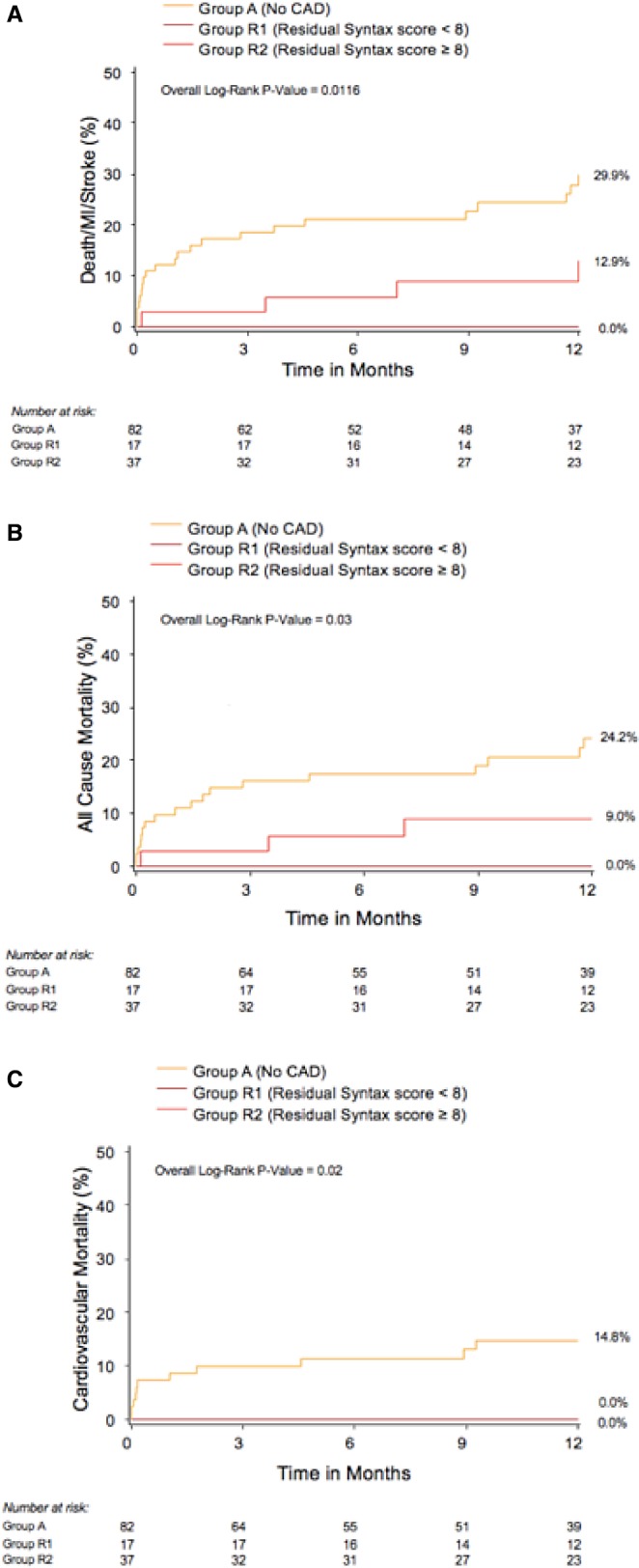
Kaplan–Meier curves at 1 year according to the residual SYNTAX score for (A) the combined end point (all‐cause death, myocardial infarction [MI], stroke); (B) all‐cause mortality; and (C) cardiovascular mortality. CAD indicates coronary artery disease.

Baseline characteristics of patients who had undergone PCI within 6 months prior to TAVR are described in Table 4. Patients with recent PCI were divided into 2 categories: low residual SS (rSS; <8) (R1) (n=17) and high rSS (≥8) (n=37) (R2). On average, PCI was performed 64.2 days prior to TAVR. As compared with those without CAD (A), patients who underwent PCI within 6 months (R1 or R2), had higher rates of hyperlipidemia, prior MI, prior PCI, prior CABG, greater logistic EuroSCORE values, lower systolic blood pressure, and lower prevalence of atrial fibrillation. Compared with the group with SS <8 (R1), patients with higher rSS (R2) had higher frequencies of prior MI (R2: 51.4% versus R1: 23.5%; *P*=0.055), prior CABG (R2: 56.8% versus R1: 5.9%; *P*=0.0004), peripheral vascular disease (R2: 35.1% versus R1: 5.9%; *P*=0.02), and higher logistic EuroSCORE (R2: 27.1% versus R1: 17.2%; *P*=0.02), and lower mean systolic blood pressure (R2: 116.7 mm Hg versus R1: 133.1 mm Hg; *P*=0.004) and peak aortic gradient (R2: 62.3 mm Hg versus R1: 76.6 mm Hg; *P*=0.0045). There was a high correlation between baseline SS and rSS (Spearman's rho=0.878, *P*<0.001), highlighting the fact that patients with greater baseline CAD severity and complexity often end up with less complete revascularization prior to TAVR (Figure 2). Patients with less complete revascularization (R2 versus R1) had similar rates of the combined primary end point (30 days: R2: 5.4% versus R1: 0% [*P*=0.33]; 1 year: R2: 10.8% versus R1: 0% [*P*=0.16]), all‐cause mortality (30 days: R2: 27.% versus R1: 0% [*P*=0.49]; 1 year: R2: 8.1% versus R1: 0% [*P*=0.23]), cardiovascular mortality (30 days: R2: 0% versus R1: 0% [*P*=NA]; 1 year: R2: 0% versus R1: 0% [*P*=NA]), MI (30 days: R2: 0% versus R1: 0% [*P*=NA]; 1 year: R2: 0% versus R1: 0% [*P*=NA]), and stroke (30 days: R2: 2.7% versus R1: 0% [*P*=0.49]; 1 year: R2: 2.7% versus R1: 0% [*P*=0.49]), at both 30 days and 1 year (Table 5 and Figure 3). Echocardiographic follow‐up at 1 year demonstrated that patients with less complete revascularization had lower LVEF (LVEF A: 60.5%; LVEF R1: 54.2%; R2: 52.5% [*P*=0.02]).

**Table 4:** Baseline Characteristics of Patients According to Residual SS

	No CAD n=82	Low Residual SS n=17	High Residual SS n=37	Total	*P* Value
Age, y	82.9	83.8	81.7	82.7	0.58
Male, %	40.7	41.2	37.8	40.0	0.95
Hypertension, %	84.1	94.1	83.8	85.3	0.55
History of smoking, %	16.7	40.0	35.5	24.6	0.15
Hyperlipidemia, %	63.4	94.1	89.2	74.3	0.002
Diabetes mellitus, %	32.9	23.5	35.1	32.4	0.69
History of CHF, %	65.9	70.6	70.3	67.6	0.86
Atrial fibrillation, %	40.2	29.4	13.5	31.6	0.014
Permanent pacemaker, %	14.6	23.5	21.6	17.6	0.52
Previous MI, %	2.4	23.5	51.4	18.4	<0.0001
Previous PCI, %	2.4	100	97.3	40.4	<0.0001
Previous CABG, %	4.9	5.9	56.8	19.1	<0.0001
Cerebrovascular disease, %	14.3	0	12.1	11.9	0.28
Peripheral vascular disease, %	25.6	5.9	35.1	25.7	0.07
COPD, %	31.7	23.5	27.0	29.4	0.74
NYHA, %					
Class II	17.1	11.8	25.0	18.5	0.44
Class III	73.2	70.6	69.4	71.9	0.91
Class IV	9.8	17.6	5.6	9.6	0.38
6MWT distance, m	169.5	151.0	157.7	163.5	0.74
Baseline creatinine, mg/dL	1.23	1.32	1.21	1.24	0.73
Hemodialysis at baseline, %	1.5	0	4.0	2.0	0.69
Mean systolic BP, mm Hg	126.9	133.1	116.7	124.9	0.006
Mean diastolic BP, mm Hg	67.2	62.8	57.9	64.1	0.002
Logistic EuroSCORE	19.8	17.2	27.1	21.5	0.014
STS risk score	8.4	8.6	10.8	9.1	0.18
SS, mean	0	15.0	35.2	17.9	<0.0001
Residual SS, mean	···	4.7	26.3	16.0	<0.0001
CABG SS, mean	···	27.0	31.7	30.4	0.62
Porcelain aorta, %	15.9	25.0	13.9	16.4	0.59
Frailty, %	32.1	56.3	30.6	34.6	0.15
Mean LVEF, %	53.7	50.4	51.6	52.7	0.60
Peak gradient, mm Hg	68.7	76.6	62.3	67.9	0.12
Mean gradient, mm Hg	42.3	46.1	38.3	4.7	0.21
Aortic valve area, cm^2^	0.62	0.56	0.64	0.62	0.35
Approach					
Transapical, %	45.1	41.2	62.2	49.3	0.18
Transfemoral, %	54.9	58.8	37.8	50.7	0.18
Valve size, %					
23 mm	56.1	35.3	54.1	52.9	0.29
26 mm	37.8	52.9	45.9	41.9	0.44
29 mm	2.4	5.9	0	2.2	0.38
Procedural success, %	88.9	100	97.3	92.6	0.12

6MWT indicates 6‐minute walk test; BP, blood pressure; CABG, coronary artery bypass graft surgery; CAD, coronary artery disease; CHF, congestive heart failure; COPD, chronic obstructive pulmonary disease; LVEF, left ventricular ejection fraction; MI, myocardial infarction; NYHA, New York Heart Association heart failure; PCI, percutaneous coronary intervention; SS, SYNTAX score; STS, Society of Thoracic Surgeons.

**Table 5:** Outcomes According to Residual SS at 30 Days and 1 Year

	No CAD	Low Residual SS	High Residual SS	Total	*P* Value
30‐d outcomes					
Combined primary end point (death, MI, stroke)	13.4	0	5.4	9.6	0.14
All‐cause mortality	9.8	0	2.7	6.6	0.18
Cardiovascular mortality	7.3	0	0	4.4	0.13
MI	4.2	0	0	2.0	0.58
Stroke	4.9	0	2.7	3.7	0.58
1‐y outcomes					
Combined primary end point (death, MI, stroke)	26.8	0	10.8	19.1	0.01
All‐cause mortality	22.0	0	8.1	15.4	0.03
Cardiovascular mortality	13.4	0	0	8.1	0.02
MI	12.5	0	0	6.0	0.18
Stroke	7.3	0	2.7	5.1	0.34
Major vascular complications	13.4	5.9	10.8	11.8	0.67
Major bleeding	8.6	0	16.2	9.6	0.15
Repeat hospitalizations	36.6	35.3	32.4	35.3	0.91
NYHA, %					
Class I	47.6	50.0	40.6	45.9	0.76
Class II	38.1	25.0	46.9	38.7	0.34
Class III	11.1	25.0	12.5	13.5	0.34
Class IV	3.2	0	0	1.8	0.46
6MWT distance, m	197.5	191.6	239.4	218	0.19

6MWT indicates 6‐minute walk test; CAD, coronary artery disease; MI, myocardial infarction; NYHA, New York Heart Association heart failure; SS, SYNTAX score.

### Impact of the CABG SS

**Figure 4:**
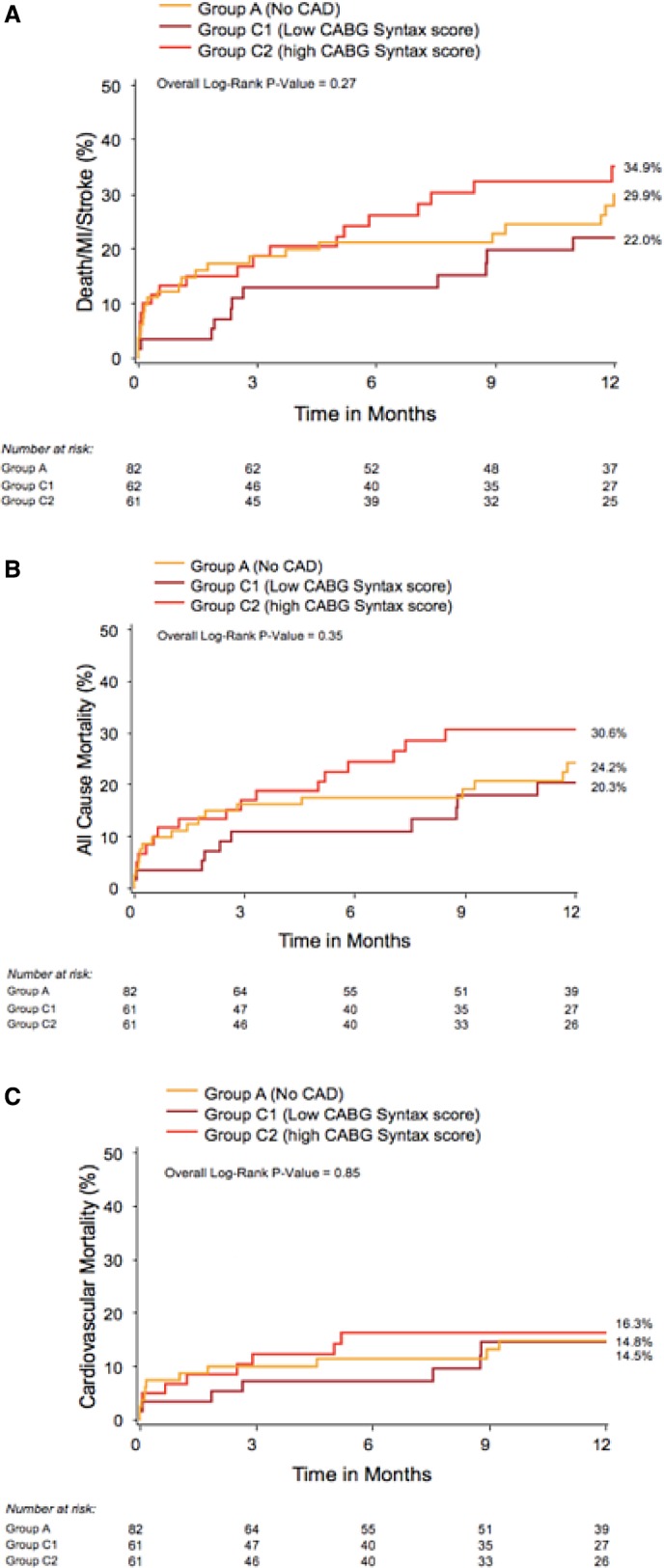
Kaplan–Meier curves at 1 year according to coronary artery bypass surgery SYNTAX score for (A) the combined end point (all‐cause death, myocardial infarction [MI], stroke); (B) all‐cause mortality; and (C) cardiovascular mortality. CABG indicates coronary artery bypass graft surgery; CAD, coronary artery disease.

Table 6 summarizes the baseline characteristics of patients with previous CABG according to the CABG SS. Compared with those with low CABG SS (C1), patients with CABG SS greater than the median CABG SS (C2) had a lower prevalence of smoking, a greater prevalence of frailty, higher logistic EuroSCORE, higher peak and mean aortic gradients, and lower aortic valve area. At 30 days, patients with a high CABG SS (C2) had a greater incidence of the combined primary end point (Table 7). At 1 year, however, although the incidence of the combined primary end point was still numerically higher in the C2 group versus the C1 group, it was not statistically significant (31.1% versus 17.7%, *P*=0.08). All other outcomes were similar between the CABG SS subgroups at 30 days and 1 year (Figure 4).

**Table 6:** Baseline Characteristics of Patients According to CABG SS

	No CAD	Low CABG SS	High CABG SS	Total	*P* Value
Age, y	82.9	80.3	82.0	81.9	0.08
Male, %	40.7	69.4	67.2	57.4	0.0005
Hypertension, %	84.1	91.9	90.2	88.3	0.31
History of smoking, %	16.7	47.6	43.4	32.9	<0.0001
Hyperlipidemia, %	63.4	95.2	93.3	81.9	<0.0001
Diabetes mellitus, %	32.9	37.1	32.8	34.1	0.84
History of CHF, %	65.9	77.4	88.3	76.0	0.017
Atrial fibrillation, %	40.2	29.5	23.0	31.9	0.08
Permanent pacemaker, %	14.6	37.1	27.9	25.4	0.008
Previous MI, %	2.4	50.0	57.4	33.2	<0.0001
Previous PCI, %	2.4	40.3	44.3	26.3	<0.0001
Cerebrovascular disease, %	14.3	12.1	24.6	16.7	0.15
Peripheral vascular disease, %	25.6	33.3	41.0	32.5	0.15
COPD, %	31.7	24.2	31.1	29.3	0.57
NYHA, %					
Class II	17.1	16.4	6.6	13.7	0.15
Class III	73.2	72.1	68.9	71.6	0.85
Class IV	9.8	11.5	24.6	14.7	0.03
6MWT, m	169.5	165.9	168	168.0	0.97
Baseline creatinine, mg/dL	1.23	1.41	1.39	1.33	0.11
Hemodialysis at baseline, %	1.5	2.3	5.6	2.8	0.48
Mean systolic BP, mm Hg	126.9	121.7	122.2	123.9	0.14
Mean diastolic BP, mm Hg	67.2	61.5	60.4	63.4	0.009
Logistic EuroSCORE	19.8	29.7	36.3	27.7	<0.0001
STS risk score	8.4	12.7	11.8	44	0.32
SS, mean	···	30.2	49.8	28.1	<0.0001
Residual SS, mean	···	17.6	38.2	29.6	0.004
CABG SS, mean	···	14.4	36.9	25.5	<0.0001
Porcelain aorta, %	15.9	10.0	3.4	10.4	0.06
Frailty, %	32.1	22.8	44.1	33.0	0.05
Mean LVEF, %	53.7	47.3	48.6	50.2	0.03
Peak gradient, mm Hg	68.7	55.6	67.5	64.4	0.0006
Mean gradient, mm Hg	42.3	32.8	41.2	39.2	0.0002
Aortic valve area, cm^2^	0.62	0.72	0.62	0.65	0.02
Approach					
Transapical, %	45.1	50.0	62.3	51.7	0.12
Transfemoral, %	54.9	50.0	37.7	48.3	0.12
Valve size, %					
23 mm	56.1	30.6	29.5	40.5	0.001
26 mm	37.8	61.3	62.3	52.2	0.003
29 mm	2.4	8.1	6.6	5.4	0.29
Procedural success, %	88.9	91.9	91.7	90.6	0.78

6MWT indicates 6‐minute walk test; BP, blood pressure; CABG, coronary artery bypass graft surgery; CAD, coronary artery disease; CHF, congestive heart failure; COPD, chronic obstructive pulmonary disease; LVEF, left ventricular ejection fraction; MI, myocardial infarction; NYHA, New York Heart Association heart failure; PCI, percutaneous coronary intervention; SS, SYNTAX score; STS, Society of Thoracic Surgeons.

**Table 7:** Outcomes According to CABG SS at 30 Days and 1 Year

	No CAD	Low CABG SS	High CABG SS	Total	*P* Value
30‐d outcomes					
Combined primary end point (death, MI, stroke)	13.4	3.2	14.8	10.7	0.07
All‐cause mortality	9.8	3.3	11.5	8.3	0.22
Cardiovascular mortality	7.3	3.3	6.6	5.9	0.58
MI	4.2	0	3.1	2.3	0.55
Stroke	4.9	0	3.3	2.9	0.23
1‐y outcomes					
Combined primary end point (death, MI, stroke)	26.8	17.7	31.1	25.4	0.22
All‐cause mortality	22.0	16.4	27.9	22.1	0.31
Cardiovascular mortality	13.4	11.5	14.8	13.2	0.87
MI	12.5	3.2	6.3	6.9	0.40
Stroke	7.3	0	3.3	3.9	0.08
Major vascular complications	13.4	3.3	6.6	8.3	0.08
Major bleeding	8.6	1.6	10.0	6.9	0.14
Repeat hospitalizations	36.6	45.9	23.0	35.3	0.03
NYHA, %					
Class I	47.6	50.0	57.1	50.7	0.66
Class II	38.1	31.8	37.1	35.9	0.79
Class III	11.1	15.9	5.7	11.3	0.36
Class IV	3.2	2.3	0	2.1	0.58
6MWT, m	197.5	261.8	272.0	230.0	0.07

6MWT indicates 6‐minute walk test; CABG, coronary artery bypass surgery; CAD, coronary artery disease; MI, myocardial infarction; NYHA, New York Heart Association heart failure; SS, SYNTAX score.

## Discussion

In this multicenter study analyzing the impact of the extent of CAD (as assessed by a core lab–validated, QCA‐derived SS) on outcomes after TAVR, we found that: (1) the presence and complexity of CAD had no influence on the incidence of the combined primary end point (all‐cause death, MI, stroke) at 30 days and 1 year after TAVR; (2) greater CAD severity was not associated with different LVEF improvement at 1 year after TAVR; (3) in patients undergoing PCI within 6 months prior to TAVR, more severe baseline CAD was associated with less complete revascularization; (4) patients with rSS >8 had similar short‐term (30 days) and midterm (1 year) clinical outcomes as compared with patients who underwent more complete revascularization by PCI (rSS <8); and (5) a high CABG SS had no association with 1‐year all‐cause mortality, cardiovascular mortality, MI, or stroke.

There are only a few conflicting published studies that have assessed the influence of concomitant CAD on procedural outcomes and long‐term survival after TAVR. In a recently published series of 445 patients, Stefanini et al^17^ demonstrated that almost two thirds of elderly patients with severe AS had evidence of CAD, that severity of CAD was related to higher risk profiles at baseline and higher adverse ischemic outcomes 1 year after TAVR, and that patients with an SS >22 had a higher risk of the composite of cardiovascular death, MI, or stroke at 1 year after TAVR, primarily due to a 2‐fold increased risk of cardiovascular death. Another retrospective study of 271 consecutive patients who underwent TAVR with the Edwards SAPIEN or SAPIEN XT transcatheter heart valves showed that patients with an SS >9 had greater 30‐day and 1‐year overall mortality than those with an SS <9.^18^ Nonetheless, dichotomized CAD status (present or absent) was not associated with lower 30‐day or 12‐month survival rates. In a study by Dewey et al,^19^ overall mortality after TAVR was significantly higher among patients with CAD (35.7% versus 18.4%, *P*=0.01).

On the other hand, in a study including 663 consecutive patients who underwent TAVR, CAD, defined as prior CABG or PCI, had no impact on all‐cause mortality (adjusted hazard ratio, 0.74; 95% CI, 0.40–1.36 [*P*=0.0331]) or major adverse cerebrovascular and cardiac events (adjusted HR, 0.76; 95% CI, 0.42–1.36 [*P*=0.353]). A recent meta‐analysis of 7 studies including 2472 patients undergoing TAVR demonstrated that, after a median follow‐up of 452 days, a diagnosis of CAD was not associated with all‐cause mortality.^20^ The results of our study corroborate the latter findings. Indeed, neither the presence nor severity of CAD were linked to all‐cause mortality, cardiovascular mortality, MI, or stroke. The rate of stroke was in fact higher in patients without CAD and this particular discovery might be caused by the higher baseline prevalence of atrial fibrillation in patients without CAD. In our study, a high proportion of patients underwent TAVR via a transapical access route (51.7%). This might have influenced the overall 30‐day mortality rate (7.7%), which is higher than the reported rates in other contemporary trials.^21^

In a study by Généreux et al,^13^ an rSS >8 was associated with poor 30‐day and 1‐year prognosis in patients with moderate‐ and high‐risk acute coronary syndromes undergoing PCI. Similarly, in a validation study of the rSS,^16^ it was demonstrated that an rSS >8 was associated with adverse long‐term clinical outcomes (including mortality) and therefore may aid in determining a reasonable level of revascularization. Intuitively, the achievement of complete revascularization seems desirable in patients undergoing revascularization prior to TAVR. However, despite significant advances in PCI, complete revascularization prior to TAVR is often not obtained. Indeed, in a single‐center cohort study of 263 elderly patients undergoing TAVR by Van Mieghem et al,^22^ complete revascularization was achieved in only 20% of TAVR patients with incomplete revascularization at baseline. Moreover, complete versus incomplete revascularization status prior to TAVR did not affect 1‐year survival (79.9% versus 77.4%, *P*=0.85).^22^ In the study by Stefanini et al,^17^ the authors obtained similar findings to our study, whereby patients with higher baseline SS received less complete revascularization as demonstrated by a higher rSS. The authors also demonstrated that an rSS >14 was linked to worse long‐term clinical outcomes after TAVR, whereas lower rSS resulted in outcomes similar to those of patients with complete revascularization. In our study, the degree of completeness of revascularization had no impact on short‐term and midterm clinical outcomes. Based on these findings, it seems that a thoughtful revascularization strategy selection by a dedicated heart team can produce favorable midterm outcome obviating the prerequisite for complete coronary revascularization.

In our cohort, individuals with higher baseline SS and those with less than complete revascularization had a lower LVEF at 1‐year follow‐up. Similarly, in a study looking at patients with significant CAD undergoing surgical aortic valve replacement with or without CABG, those who did not receive complete revascularization experienced more left ventricular dysfunction postoperatively.^23^

Robust randomized data are lacking regarding both the management and the impact of CAD in patients undergoing TAVR. Conducted at a number of European centers, ACTIVATION (Percutaneous Coronary Intervention Prior to Transcatheter Aortic Valve Implantation: A Randomized Controlled Trial) is the first randomized study to specifically evaluate the influence of coronary revascularization by PCI for significant CAD (proximal coronary stenosis ≥70%) prior to TAVR. The trial analyzes the hypothesis that a strategy of pre‐TAVR PCI is noninferior to TAVR without revascularization, with a 12‐month composite primary outcome of mortality and rehospitalization. Hopefully, a clearer understanding of the best management strategy will emerge.

### Study Limitations

In this study, baseline and clinical end points were prospectively collected, yet the extent and complexity of CAD were evaluated by retrospectively calculating the SS. In addition, the SS, rSS, and CABG SSs were assessed by interventional cardiologists and core lab technicians with QCA analysis in whom good reproducibility has been demonstrated. However, the SS is based purely on angiographic interpretation that has obvious limitations. The results of our study may have been different if the SS was assessed differently (eg, less trained readers). Another limitation is that neither the reproducibility nor the prognostic role of the CABG SS have been validated in large studies.^24^ Although our study is one of the largest series on the impact of severity and complexity of CAD prior to TAVR, the number of patients in our analysis was relatively small. The analyses of the rSS and the CABG SS were underpowered and subject to a type II statistical error. Since many of the patients who received TAVR in our institutions came from numerous referring centers, it has not been possible to gather all coronary angiograms for detailed core lab analysis. Moreover, in this study, the 1‐year results provide insights into the influence of CAD in the midterm but prevent extrapolation to longer‐term follow‐up.

Although the outcomes in each center were defined according strict definitions, not all events were adjudicated by an adjudication committee.

Although multivariable analysis was performed for significant confounders, the possibility of other unmeasured confounders to have affected the results cannot be excluded. For all these reasons, the results of this report should probably be considered hypothesis generating. Prospective randomized trials are needed to clarify the impact of the extent of CAD on clinical outcomes following TAVR.

## Conclusions

In our mutlicenter, core lab–validated study, neither the severity of CAD nor completeness of revascularization after PCI or CABG as assessed by QCA‐derived SS, rSS, and CABG SS, were associated with clinical outcomes after TAVR, at both 30 days and 1 year. Considering the worldwide trend of treating lower‐risk patients with TAVR, further randomized studies are needed to clarify the impact of CAD on TAVR outcomes.

## Disclosures

DeLarochellière has received consulting fees from St. Jude Medical. Dumont has received consulting fees from Edwards Lifesciences. Kodali is a medical advisory board member of Paieon Medical; has received consulting fees from Edwards Lifesciences, Medtronic, and St. Jude Medical; and owns stock options in Thubrikar Aortic Valve. Leon is a nonpaid member of the scientific advisory board of Edwards Lifesciences and Medtronic Vascular. Rodés‐Cabau has received consulting fees or research grants from Edwards Lifesciences, Medtronic, and St. Jude Medical. The other authors report no conflicts.
